# Shared decision making with Oncologists and Palliative care specialists (SOP) model help advanced pancreatic cancer patients reaching goal concordant care: A prospective cohort study

**DOI:** 10.1002/cam4.6590

**Published:** 2023-09-23

**Authors:** Yung‐Ling Tseng, Yun‐Ching Lin, Wan‐Ju Hsu, Ya‐Chun Kang, Hsin‐Yin Su, Shao‐Yi Cheng, Jaw‐Shiun Tsai, Tai‐Yuan Chiu, Hsien‐Liang Huang

**Affiliations:** ^1^ Department of Education Kuang Tien General Hospital Taichung Taiwan; ^2^ Cancer Administration and Coordination center National Taiwan University Hospital Taipei city Taiwan; ^3^ Department of Nursing National Taiwan University Hospital Taipei city Taiwan; ^4^ Department of Family Medicine National Taiwan University Hospital Taipei city Taiwan

**Keywords:** emergency room visit, hospital admission, intensive care unit admission, palliative care, pancreatic cancer, shared decision‐making

## Abstract

**Background:**

Pancreatic cancer is often diagnosed at a late stage with a poor prognosis due to insidious symptoms and lack of evidence‐based screening in general population. Palliative care's acceptance in Asian cultures is hindered by misconceptions and ineffective communication about management that improve quality of life other than cancer directed treatment. Our study aimed to determine the effect of the Shared decision‐making with Oncologists and Palliative care specialists (SOP) model developed from the traditional shared decision‐making (SDM) model on the palliative care acceptance rate and medical resource utilization.

**Methods:**

This is a prospective cohort study implementing the SOP model at the National Taiwan University Hospital from January 2018 to December 2019 for patients with advanced pancreatic cancer. Medical resource utilization was defined and recorded as the rate of hospitalization, emergency room (ER), and intensive care unit admissions. We compared the results between two groups: patients who received the SOP model in 2019 and patients who did not receive it in 2018.

**Results:**

137 patients with advanced pancreatic cancer were included in our study. The result showed that the acceptance rate of palliative care significantly increased from 50% to 78.69% after the SOP model (*p* = 0.01). The hospitalization rate did not show a significant difference between 2018 (93.42%, 95% CI: 0.88–0.99) and 2019 (93.44%, 95% CI: 0.87–1.00). 83.61% (95% CI: 0.74–0.93) of our patients in 2019 had at least one ER visit; the rate was 81.5% (95% CI: 0.73–0.91) in 2018 (*p* = 0.28). The percentage of patients admitted to the ICU increased from 3.95% in 2018 to 8.2% (95% CI: −0.05–0.08) in 2019 (95% CI: 0.11–0.15) (*p* = 0.00). The hospitalization and ER visit showed no statistically difference between 2 years.

**Conclusions:**

The modified SOP model markedly augmented palliative care's acceptance of patients with advanced pancreatic cancer. Adoption of the SOP model would provide these patients a more proactive and systematic approach to deliver needed healthcare.

## 1 INTRODUCTION

Pancreatic cancer is an aggressive cancer, as the diagnosis is generally made very late and the therapeutic treatment options become very limited compared to other advanced diseases.^1^ Metastatic pancreatic cancer remains one of the most challenging cancers to treat, with a poor prognosis and a median survival time of only 11 months, despite recent therapeutic developments using newer strategies such as genomics, stromal therapies, and immunotherapies.^2^ The overall 5‐year survival rate is 5%.^3^ Considering the fast progression pancreatic cancer, palliative care is essential to support patients and their families.^4^

In standard cancer care, the oncologist assumes a pivotal role in leading the treatment decision‐making process, involving cancer‐directed treatment like surgery, radiation therapy, chemotherapy, targeted therapy, and immunotherapy. On the other hand, palliative care offers comprehensive support to patients diagnosed with serious illnesses, with the goal of enhancing the quality of life for both patients and their families. It encompasses the management of physical and psychological symptoms, along with addressing social and spiritual aspects of care, administered by a multidisciplinary palliative care team in both inpatient and outpatient settings.^5^ Compared to palliative care, end‐of‐life (EOL) care specifically pertains to the final months, weeks, or days of a patient's life. During this phase, cancer‐directed treatments are typically ceased, and the focus shifts solely to alleviating symptoms.

Building efficient patient‐centered communication with patients and introducing the concept of palliative care early is important, as accepting early palliative care may improve the outcome of oncology patients.^5^ Several studies have demonstrated that palliative care in the advanced cancer stage improves quality of life, controls physical symptoms, enhances patients' spiritual well‐being, and provides more effective allocation of medical resource utilization.^5^, ^6^ However, a critical issue has been discussed intensely in recent years regarding the low rate of enrollment in palliative care. According to the World Health Organization, the current situation is far from ideal.^7^ Only about 14% of population in need receives palliative care, while the global need continues to grow. Besides overly restrictive policies on palliative medicine in some countries, misconceptions about palliative care remain a primary barrier to its use. Another important factor is the lack of effective communication between patients and physicians about available palliative care services.^8^ As patients rarely express willingness to choose palliative care at the time of diagnosis due to lack of information, how to ameliorate the situation has become a critical issue.^9^

Evidence has suggested the role of shared decision‐making (SDM) in helping patients and their caregivers understand and adapt palliative care.^10^ SDM is a collaborative mode between patients, caregivers, and clinicians in making clinical healthcare plans together by providing information, elaborating on concerns, and building a patient‐preferred goal of treatment.^11^ Involving patients in making choices for their own care plan is related to augmentation of human dignity and enhancement of patients' satisfaction and compliance.^12^ Different tools and strategies other than face‐to‐face talk used in the decision process aid patients to be involved and are more likely to encourage patients to express their preferences on healthcare choices. Given the wide range of personal values and preferences that can impact treatment decisions, it is crucial to allocate sufficient time to the communication process. This allows clinicians to thoroughly assess the attitudes of patients and their families regarding living with life‐limiting illness and associated mortality, facilitating the development of patient‐centric strategies.^13^ Regular SDM meetings might be more reasonable as patients' decisions for treatment vary widely secondary to the harm versus benefits and the waning of health status. However, the absence of standardized guidelines for initiating palliative care suggests a lack of clarity or consistency in the approach to communicating the concept of palliative care to patients. The method of communication and how this information of palliative care is delivered to patients are shown to affect patients' outcomes, as well as their behavioral changes related to the rates of visiting medical institutes.^14^, ^15^

Detailed information about the benefits and drawbacks of treatment options provided by the mixed mode of SDM helps patients to picture a realistic expectation and appears to lower the barrier for accepting palliative care service.^16^ A clear SDM intervention is crucial to support terminally ill patients in making truly informed autonomous decisions for their EOL care, including do not resuscitate (DNR) and advanced care planning.

Previous studies have demonstrated significant benefits from early integration of interdisciplinary and supportive care, including palliative care, into the treatment of pancreatic cancer. Such benefits may include improvements in quality of life, reduced rates of depression, and decreased symptom burden.^1^, ^17^, ^18^ However, the optimal delivery model of these supportive services during the time of rapid advancement with cancer‐directed treatment is needed. Furthermore, the model must be based on an SDM base to fit patient and family goals of care. Based on this background, the Shared decision‐making with Oncologists and Palliative care specialists (SOP) model, which integrates the essential of SDM into clinical practice, was developed.^19^ Our study aims to evaluate the influence of early integration of palliative care concepts including optimal symptom management and function improvement with palliative care within the SOP model, on the acceptance rate of palliative care among Taiwanese individuals diagnosed with pancreatic cancer. This investigation was motivated by the common practice of introducing palliative care late in the disease trajectory in Taiwan due to misconception, which is recognized as suboptimal for effective decision‐making processes. Previous research has not specifically examined palliative care acceptance in the Asian population with pancreatic cancer. The findings of our study may apply to other patients with advanced cancer who are undergoing cancer‐directed treatments.

## 2 METHODS

### 2.1 Study design and participants

**FIGURE 1:**
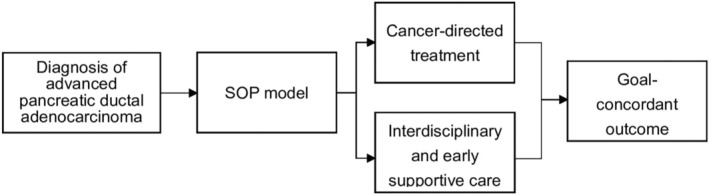
Shared decision‐making with Oncologist and Palliative care specialists (SOP) model for patients with advanced pancreatic ductal adenocarcinoma.

**FIGURE 2:**
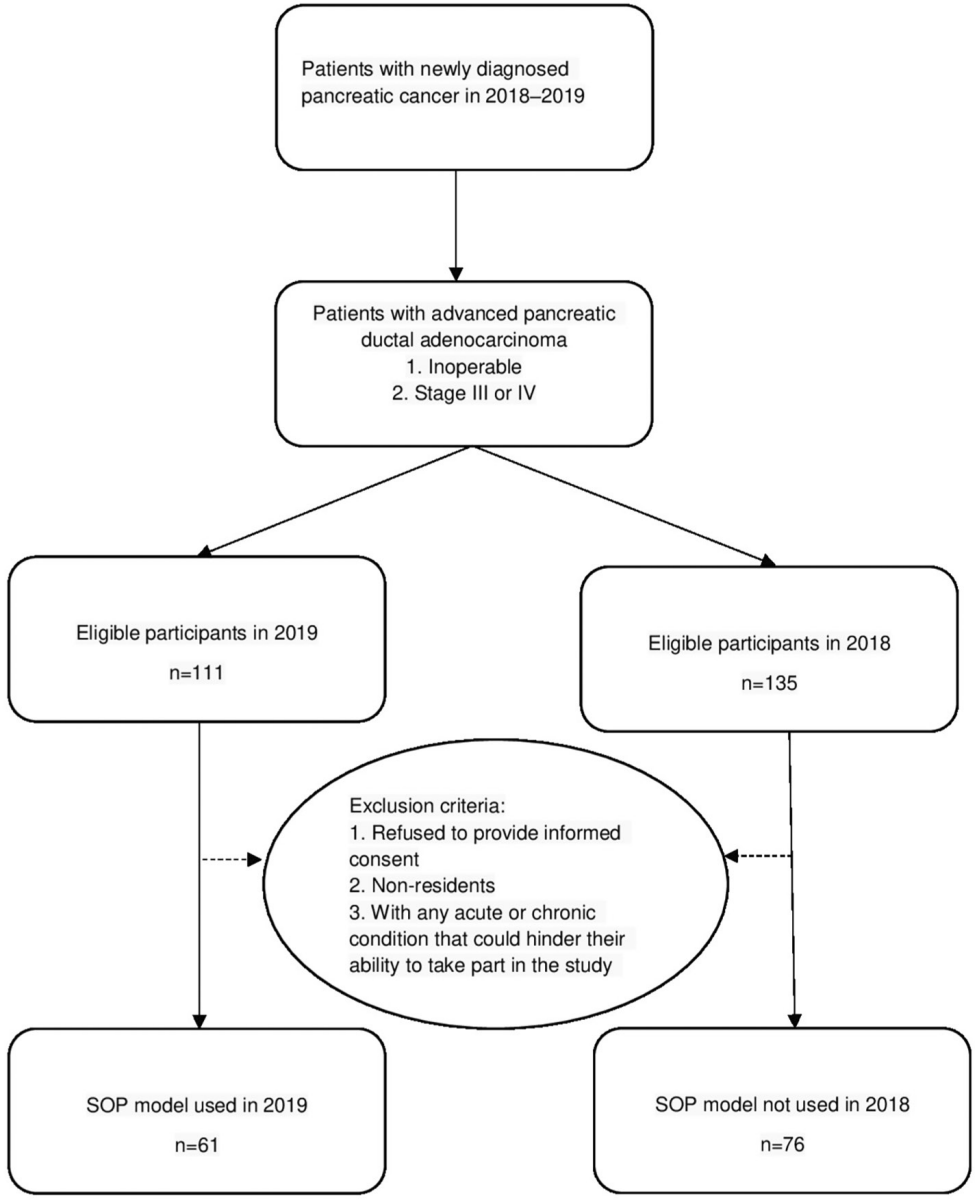
Flow chart of inclusion criteria and exclusion criteria.

This prospective cohort study was conducted at a national cancer treatment medical center oncology outpatient clinic between January 2018 and December 2019. Patients were eligible to participate if they were newly diagnosed with pancreatic ductal adenocarcinoma, unresectable advanced disease, Stage III or IV disease, were >20 years of age, and were able to read and respond to the decision aid provided in the SOP model. Patients who refused to provide informed consent, non‐residents, and those with any acute or chronic condition that could hinder their ability to take part in the study were excluded from our research. The process of joining SOP model is shown in Figure 1. The recruitment process for patients in our study is illustrated in Figure 2.

### 2.2 Model assignment and development

The SOP model was implemented in 2019, and patients in 2018 received usual oncology care with case managers' follow‐up according to the cancer treatment schedule only.

We developed the SOP model used in this study in 2016 based on the integration of the traditional three‐talk model of SDM and the SHARE model (Seek participation, Help comparison, Assess values, Reach decision, Evaluate decision), proposed by the Agency for Healthcare Research and Quality. The design of the SOP model guides communication among patients, families, and healthcare providers, enabling them to make informed decisions about treatment and care. The detailed framework can be found in our previous publication.^19^, ^20^ The SOP model was subdivided into three steps, according to the traditional SDM model: *choice talk*, *option talk*, and *decision talk*.^11^ *Choice talk* was designed for oncologists to demonstrate all the possibilities regarding treatment and care to patients with advanced pancreatic cancer and their families, providing a brief frame of the treatment plan. It is important to initiate exploration of patients' preference for treatment, the choice and whereabouts for EOL care, concept of palliative care, and DNR in this step for the preparation of the upcoming *option talk* step. If patients were willing to join the SOP model after preliminary assessment in oncology outpatient clinics, they were referred to palliative specialists in the Family Medicine Department for further communication in *option talk* and *decision talk*. A pamphlet, designed as a decision support tool, was used to assist in decision‐making. The pamphlet was created according to the Grade of Recommendations Assessment, Development, and Evaluation regarding ethics, quality of care, and evidence‐based medicine.^21^, ^22^ The goal of *option talk* was to clarify all the benefits and harms regarding each treatment and alternatives to patients for the condition.^23^ The communication aimed first to build rapport between patients and physicians, second to address symptoms, and adverse effects caused by current anti‐cancer treatment, and third to explore patients' EOL care preferences. The final decision will be made based on the patients' preferences in the *decision talk* step.

### 2.3 Outcome measures

In our study, the main outcome of interest was the acceptance rate of palliative care whether receiving concurrently with anti‐cancer treatments or not, defined as supportive care provided by a multidisciplinary team, including palliative specialists, nutritionists, social workers, clinical psychologists, Buddhist chaplains, and volunteers. The care can be delivered as home care, shared care, (receiving palliative service at wards other than palliative ward) and palliative unit admission.^24^ Patients who willingly accepted the comprehensive care delivered by a multidisciplinary team were categorized as having accepted palliative care. However, patients seeking only specific services were not included in the recorded count of accepted palliative care. The other three outcomes included hospital admission, emergency room (ER) visits, and intensive care unit admissions 1 month before death. The decision to choose “usual care” refers to the treatment approach that differs from the SOP model. This approach involves the oncologist primarily leading the decision‐making process, which may include cancer‐directed options such as surgery, radiation therapy, chemotherapy, targeted therapy, and immunotherapy, as well as symptom control therapy. These treatments are partially covered by the national health welfare program.

### 2.4 Ethics statement

The study was performed in accordance with the principles of the Declaration of Helsinki. The hospital's institutional review board approved the study protocol, and patients who agreed to follow‐up with model implementation provided consent to participate in the study. Our study was approved by National Taiwan University Hospital Research Ethics Committee (IRB number 201901144RINB).

### 2.5 Statistical analysis

All categorical variables in the study are expressed as proportions and were analyzed using the chi‐squared test and Fisher exact test, if applicable. The demographic data were analyzed using the independent *t*‐test. Statistical significance was set at *p* < 0.05. All statistical analyses were performed using the SPSS ver 21.0 software package (IBM Corp.).

## 3 RESULTS

### 3.1 Demographic and clinical characteristics

Our study, conducted between 2018 and 2019, initially included 246 patients with advanced pancreatic cancer. However, due to loss of follow‐up, we were only able to include data from 137 patients in our final analysis. A total of 137 patients were included in the final analysis, with loss of follow‐up of those who were not willing to participate in the study, discontinued treatment with loss of follow‐up, or had incomplete data. In 2019, 61 patients joined the SOP model, and in 2018, 76 patients did not.

The baseline characteristics of the study participants are shown in Table 1. The median age was 68.4 years with a relatively balanced sex proportion. (Male: Female ratio = 6:4). The educational status of the sample was at two extremes: 29.9% of the patients had an educational level in elementary school or below, while the other 17% signified their educational status as university or above. The majority of patients were married (78.8%), while the others were widowed (10.2%), single (4.4%), or divorced (4.4%). Among the patients, 33 (24.1%) were rated as having an Eastern Cooperative Oncology Group (ECOG) performance status 1, 18 (13.1%) as performance status 2, 28 (20.4%) as performance status 3, and 68 (49.6%) as performance status 4. ECOG performance 3 and above mean that the patients had only limited self‐care ability, were confined to bed for >50% of waking hours, and were not suitable for aggressive chemical treatment. The chi‐squared analysis showed the homogeneity of the two groups of patients admitted in 2018 and 2019 (Table 1).

**TABLE 1:** Demographic characteristics of patients (*n* = 137).

	PC overall	PC annual data report		
		2018	2019	
Clinical characteristic	(*n* = 137)	(*n* = 76)	(*n* = 61)	
Age, years	68.4	69	67.5	0.000*
Sex				
Male	82 (59.9)	47 (61.8)	35 (57.4)	
Female	55 (40.1)	29 (38.2)	26 (42.6)	
Education				0.365
Elementary school or below	41 (29.9)	22 (28.9)	19 (31.1)	
Junior high school	17 (12.4)	11 (14.5)	6 (9.8)	
Senior high school	27 (19.7)	13 (17.1)	14 (23)	
Junior college	12 (8.8)	12 (15.8)	0 (0.0)	
University or above	23 (16.8)	0 (0.0)	22 (36.1)	
Other or unknown	0 (0.0)	0 (0.0)	0 (0.0)	
Marital status				0.848
Married	108 (78.8)	56 (73.7)	52 (85.2)	
Widowed	14 (10.2)	9 (11.8)	5 (8.2)	
Single	6 (4.4)	5 (6.6)	1 (1.6)	
Divorced/Separated	6 (4.4)	4 (5.3)	2 (3.3)	
Unknown	2 (1.5)	2 (2.6)	0 (0.0)	
Metastasis				0.672
Without	24 (17.5)	18 (23.7)	6 (9.8)	
With	110 (80.3)	56 (73.7)	54 (88.5)	
Unknown or multiple sites	3 (2.2)	2 (2.6)	1 (1.6)	
ECOG performance status				0.28
1	33 (24.1)	11 (14.5)	12 (19.7)	
2	18 (13.1)	6 (33.3)	12 (66.6)	
3	28 (20.4)	19 (25.0)	9 (14.8)	
4	68 (49.6)	40 (52.6)	28 (45.9)	
Religion				0.43
Taoism/Traditional religions	40 (29.2)	18 (23.7)	22 (36.1)	
Buddhism	45 (32.8)	26 (34.2)	19 (31.7)	
Christianity	8 (5.8)	5 (6.6)	3 (4.9)	
Not specified	44 (32.1)	27 (35.5)	17 (27.9)	
Other	0 (0.0)	0 (0.0)	0 (0.0)	

*Note*: Data are presented as *n* (%).

Abbreviations: DNR, do not resuscitate; ECOG, Eastern Cooperative Oncology Group; PC, pancreatic cancer.

* *p* < 0.05.

**FIGURE 3:**
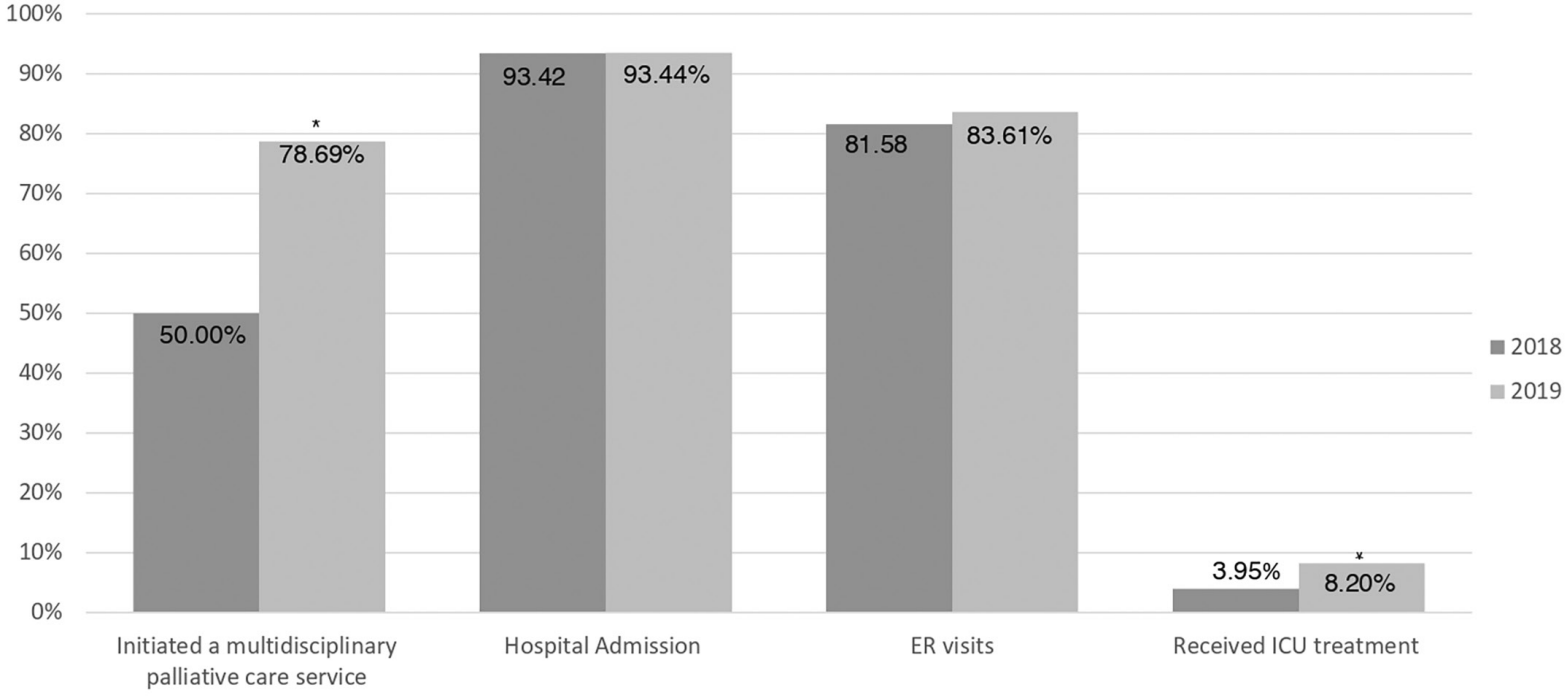
Comparison of medical treatment requested by patients with pancreatic cancer between 2018 and 2019. *p < 0.05. ER, emergency room; ICU, intensive care unit.

Our data demonstrated that patients' willingness to receive palliative care significantly increased from 50% in 2018 to 78.69% 2019 after implementing the SOP model in the treatment discussion process (*p* = 0.007, chi‐squared test). The hospitalization rate did not show a significant difference between 2018 (93.42%, 95% CI: 0.8772–0.9912) and 2019 (93.44%, 95% CI: 0.8705–0.9983) (p = 0.454). Overall, 83.61% (95% CI: 0.7405–0.9317) of our patients had one ER visit at least in 2019; the rate was 81.5% (95% CI: 0.7266–0.9050) in 2018 (*p* = 0.28). The percentage of patients admitted to the ICU increased from 3.95% in 2018 to 8.2% (95% CI: −0.053–0.0843) in 2019 (95% CI: 0.111–0.1528) (*p* = 0.000) (Figure 3).

## 4 DISCUSSION

The SOP model provides an innovative approach for the early integration of palliative care and effectively increases the rate of initiating multidisciplinary palliative care instead of standardized cancer‐modifying treatment in patients with advanced pancreatic cancer. Incorporating the SOP model into EOL care discussions with patients markedly enhanced the palliative enrollment rate: from 50% to 78.69%. Medical resource utilization, such as hospital admissions and ER visits, was not significantly different between the usual care and SOP model patients. The results of our study indicate that the integration of multidisciplinary palliative care into the SOP model had a significant impact on patients' attitudes towards palliative EOL care. However, we also observed that patients experiencing symptoms still tended to seek hospital admission to alleviate their discomfort.

In the past, patients were assumed to start aggressive cancer‐directed treatment as soon as the diagnosis was made and had few chances to understand palliative care as part of management.^23^ It is noteworthy that the traditional attitude held by caregivers towards the patient in their cultures may be a strong emphasis on the importance of pursuing cancer‐directed treatment for cancer, and patients and their families may be reluctant to consider the option of palliative care, as it may be seen as giving up or accepting defeat.^11^, ^25^ Furthermore, the negative connotation of palliative care equals giving up life, potentially causing hesitation of patients and their caregivers.^26^ The SOP model invited patients and their caregivers to share the decision‐making steps instead of relying solely on expert decisions. Joining the model not only revealed every possible care mode with risks and benefits but also provided opportunities for patients to express preferences and more time for patients to deliberate their care options. Recent literature has shown the benefits of sufficient time in the decision‐making process.^27^ We have found that utilizing our three‐phase SOP model, decision‐aided pamphlet, and palliative specialists, resulted in patients preferring multi‐disciplinary palliative care services in EOL care, demonstrating the positive impact of effective communication. These results are consistent with those of a previous randomized controlled trial.^28^ Other studies have also shown that clear information and communication lead to a more positive attitude towards palliative care and earlier hospice enrollment.^29^, ^30^

Hospital admissions, ER visits, and ICU treatment are stressful events that decrease the health‐related quality of life of patients with advanced disease.^31^ Although a recent study reported an association between palliative care and less medical resource utilization,^32^ we did not find the same outcome in our study. First, patients and their families depend on the inpatient setting to control the increased symptom burden.^33^ Pain and disease progression were indicated as the main reasons for unexpected readmission.^34^, ^35^ A review of randomized trials revealed no change in the reduction of medical costs and resource utilization in palliative care.^36^ Not surprisingly, hospital readmission was seen across studies in different disease trajectories.^37^, ^38^ The aim of ER, ICU and hospital admission of the patients was supportive rather than cancer‐directed.

Due to advances in cancer management and prognosis, there has been a shift in the attitude of cancer patients towards admission to the ICU over time.^39^ Adequate medical support, whether inpatient or home‐based, is an essential element to decrease the mental burden of caregivers and to help stabilize patients' condition.^40^, ^41^ When patients experience symptoms caused by the disease itself or complication of treatment, they may require receiving immediate medical attention. In fact, it has been reported that 33% of patients with pancreatic cancer require admission to the ICU at some point during their disease trajectory.^42^ The results of another study indicated that early palliative care referral had no impact on the ICU admission rate,^43^ which is consistent with our findings. Although palliative care may not significantly affect medical service utilization, integrating multidisciplinary medical support into palliative care plans alongside cancer treatment plans can still lead to improved quality of care.

Last but not least, the comprehensive data provided by our SOP model may contribute to decision‐making hesitancy, potentially causing patients to seek second opinions elsewhere, and ultimately reverting to specialist‐directed decisions. Furthermore, the SOP model may initially burden cancer patients and clinicians with substantial information, exacerbating existing stress, and posing challenges in adapting to the new approach. Nevertheless, by incorporating SDM, our model has the potential to improve patient satisfaction, increase treatment adherence, and ultimately lead to better health outcomes over time.

### 4.1 Limitations

Our study has some limitations. First, the study had small numbers of participants; therefore, a larger number of cases and multicenter cooperation need to be conducted to further prove our results. However, this was a pilot testing study in nature, and the current SDM model already revealed the potential to implement the model in other settings. Our study was not randomized, which is a limitation to be considered. Ideally, the proposed model should be compared with other models in a population selected from 2019. However, due to limitations in the number of cases available for our study, we were only able to perform our research between 2018 and 2019. Due to the lack of detailed information regarding the timing of therapy administration prior to death, we were unable to provide a more comprehensive explanation for the ICU admission rate. While we posited that physical symptoms could potentially account for hospital admissions, regrettably, we did not document the specific reasons for admission within our study. Moreover, COVID‐19 largely changed preferences and care style of patients and their family. Home care may gain more attention and will be a trending topic in recent years. Psychological impact on decision making caused by pandemic is worthy of further research. Moreover, we did not integrate patient report outcome into our study. As the feedback signified the satisfaction from patients, we can improve the whole process according to the information provided.

## 5 CONCLUSIONS

The SOP model markedly augmented the rate of initiating multidisciplinary palliative care in patients with advanced pancreatic cancer. This SDM process provides patients with multiple perspectives according to their complexities and needs. We encourage adoption of the SOP model in different types of cancer management to have a more proactive and systematic approach to deliver needed healthcare. The model focusing on patient‐centered decisions will be useful in the oncology field to guide specialists, patients, and their families to reach ideal goal‐concordant care.

## AUTHOR CONTRIBUTIONS

**Yung‐Ling Tseng:** Data curation (equal); formal analysis (lead); writing – original draft (lead); writing – review and editing (equal). **Yun‐Ching Lin:** Resources (equal). **Wan‐Ju Hsu:** Resources (equal). **Ya‐Chun Kang:** Resources (equal). **Hsin‐Yin Su:** Resources (equal). **Shao‐Yi Cheng:** Resources (equal). **Jaw‐Shiun Tsai:** Resources (equal). **Tai‐Yuan Chiu:** Resources (equal). **Hsien‐Liang Huang:** Conceptualization (lead); data curation (equal); investigation (lead); methodology (lead); project administration (lead); resources (lead); supervision (lead); writing – review and editing (equal).

## FUNDING INFORMATION

The authors declare there is no funding for this study.

## CONFLICT OF INTEREST STATEMENT

The authors whose names are listed certify that they have no affiliation with or involvement in any organization or entity with nay financial interest, or non‐financial interest in the subject matter or materials discussed in this manuscript.

## Data Availability

The datasets generated during and/or analyzed during the current study are available from the corresponding author on reasonable request.
